# Vascular histopathology and connective tissue ultrastructure in spontaneous coronary artery dissection: pathophysiological and clinical implications

**DOI:** 10.1093/cvr/cvab183

**Published:** 2021-05-28

**Authors:** Marios Margaritis, Francesca Saini, Ania A Baranowska-Clarke, Sarah Parsons, Aryan Vink, Charley Budgeon, Natalie Allcock, Bart E Wagner, Nilesh J Samani, Jan von der Thüsen, Jan Lukas Robertus, Mary N Sheppard, David Adlam

**Affiliations:** Department of Cardiovascular Sciences and National Institute for Health Research Leicester Biomedical Research Centre, Glenfield Hospital, Groby Road, Leicester LE3 9QP, UK; Department of Cardiovascular Sciences and National Institute for Health Research Leicester Biomedical Research Centre, Glenfield Hospital, Groby Road, Leicester LE3 9QP, UK; Department of Cardiovascular Sciences and National Institute for Health Research Leicester Biomedical Research Centre, Glenfield Hospital, Groby Road, Leicester LE3 9QP, UK; Department of Forensic Medicine, Victorian Institute of Forensic Medicine, Monash University, Melbourne, VIC, Australia; Department of Pathology, University Medical Center Utrecht, Utrecht University, Utrecht, The Netherlands; Department of Cardiovascular Sciences and National Institute for Health Research Leicester Biomedical Research Centre, Glenfield Hospital, Groby Road, Leicester LE3 9QP, UK; School of Population and Global Health, University of Western Australia, Perth, WA 6009, Australia; Department of Cardiovascular Sciences and National Institute for Health Research Leicester Biomedical Research Centre, Glenfield Hospital, Groby Road, Leicester LE3 9QP, UK; Core Biotechnology Services, College of Life Sciences, University of Leicester, LE1 7JA, UK; Electron Microscopy, Histopathology Department, Royal Hallamshire Hospital, Sheffield Teaching Hospitals, Sheffield S10 2JF, UK; Department of Cardiovascular Sciences and National Institute for Health Research Leicester Biomedical Research Centre, Glenfield Hospital, Groby Road, Leicester LE3 9QP, UK; Department of Pathology, Erasmus MC, University Medical Center Rotterdam, PO Box 2040, 3000 CA, Rotterdam, The Netherlands; Department of Pathology, Royal Brompton Hospital, London, SW3 6NP, UK; National Heart & Lung Institute, Imperial College London, London, SW3 6LY, UK; CRY Department of Cardiovascular Pathology, Molecular and Clinical Sciences Research Institute, St Georges Medical School, London SW17 0RE, UK; Department of Cardiovascular Sciences and National Institute for Health Research Leicester Biomedical Research Centre, Glenfield Hospital, Groby Road, Leicester LE3 9QP, UK

**Keywords:** Spontaneous coronary artery dissection, Autopsy, Inflammation, Collagen, Sudden cardiac death, Vascular, Electron microscopy, Haematoma

## Abstract

**Aims:**

Spontaneous coronary artery dissection (SCAD) is a cause of acute coronary syndromes and in rare cases sudden cardiac death (SCD). Connective tissue abnormalities, coronary inflammation, increased coronary *vasa vasorum* (VV) density, and coronary fibromuscular dysplasia have all been implicated in the pathophysiology of SCAD but have not previously been systematically assessed. We designed a study to investigate the coronary histological and dermal collagen ultrastructural findings in SCAD.

**Methods and results:**

Thirty-six autopsy SCAD cases were compared with 359 SCAD survivors. Coronary and myocardial histology and immunohistochemistry were undertaken. Transmission electron microscopy (TEM) of dermal extracellular matrix (ECM) components of *n* = 31 SCAD survivors and *n* = 16 healthy volunteers were compared. Autopsy cases were more likely male (19% vs. 5%; *P* = 0.0004) with greater proximal left coronary involvement (56% vs. 18%; *P* < 0.0001) compared to SCAD survivors. *N* = 24 (66%) of cases showed no myocardial infarction on macro- or microscopic examination consistent with arrhythmogenic death. There was significantly (*P* < 0.001) higher inflammation in cases with delayed-onset death vs. sudden death and significantly more inflammation surrounding the dissected vs. non-dissected vessel segments. *N* = 17 (47%) cases showed limited intimal fibro-elastic thickening but no features of fibromuscular dysplasia and no endothelial or internal elastic lamina abnormalities. There were no differences in VV density between SCAD and control cases. TEM revealed no general ultrastructural differences in ECM components or markers of fibroblast metabolic activity.

**Conclusions:**

Assessment of SCD requires careful exclusion of SCAD, particularly in cases without myocardial necrosis. Peri-coronary inflammation in SCAD is distinct from vasculitides and likely a reaction to, rather than a cause for SCAD. Coronary fibromuscular dysplasia or increased VV density does not appear pathophysiologically important. Dermal connective tissue changes are not common in SCAD survivors.

## Graphical Abstract

**Graphical Abstract:**
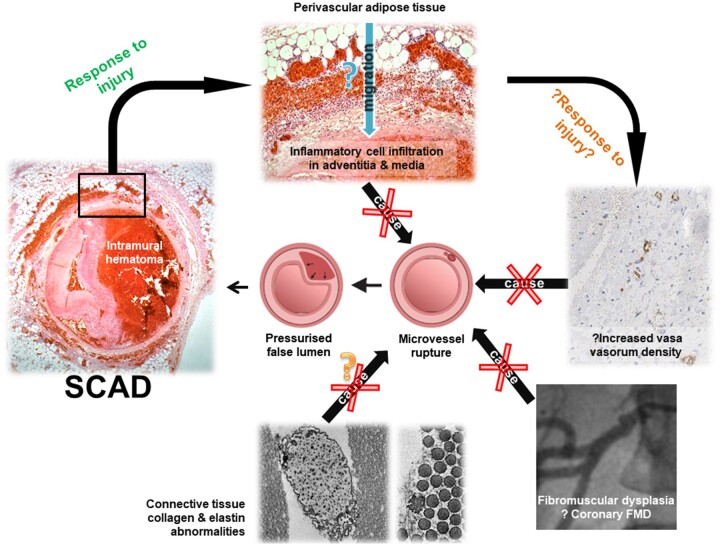

**See the editorial comment for this article ‘Is there more than meets the eye? Spontaneous coronary artery dissection and sudden cardiac death’, by John R. Giudicessi and Sharonne N. Hayes, https://doi.org/10.1093/cvr/cvac043.**

**Translational Perspective**

Spontaneous coronary artery dissection (SCAD), especially of distal coronary territories should be carefully assessed at post-mortem in SCD cases, even where there are no signs of myocardial infarction. The immediate cause of SCAD is likely to be the development of a spontaneous intramural haematoma rather than an intimal disruption or ‘tear’. This does not seem to be directly related to increased VV density, coronary fibromuscular dysplasia, or local inflammation (except as a response to injury). Although SCAD is rarely associated with hereditary connective tissue disorders, there does not seem to be a more generalizable global connective tissue ultrastructural abnormality in most cases.

## 1. Introduction

Spontaneous coronary artery dissection (SCAD) is an uncommon cause of acute coronary syndromes. Clinical presentation is usually with acute myocardial infarction, which may be associated with ventricular arrhythmia in 3–10% of cases.^1,2^ In rare cases, the first clinical manifestation is with sudden cardiac death (SCD). These cases will present at autopsy although diagnosis can be challenging and SCAD may be under-represented in the autopsy series of SCD.^3^

Descriptions of the range of histopathological findings in SCAD have been limited to isolated case reports and small series. SCAD is reportedly characterized by the presence of an intramural thrombus within a false lumen in the tunica media of the affected coronary artery.^3^ This leads to a longitudinal dissection plane causing external compression of the true lumen. Two mechanistic hypotheses have been proposed. The inside-out hypothesis suggests an endothelial-intimal disruption (‘dissection flap’) as the primary event allowing blood to enter the sub-intimal space, whereas the outside-in hypothesis suggests the primary event is a *de novo* intramural bleed.^1,2,4^ One clinical imaging study reported an increase in vasa vasorum (VV) density as a potential source for an intramural bleed,^5^ although a subsequent larger study did not replicate this finding.^6^ A role for inflammation^7,8^ and abnormalities of connective tissue^9^ in the pathophysiology of SCAD has also been proposed. Indeed, inflammatory cell infiltration of the peri-adventitial tissue surrounding the affected coronary has often been described as a component of the histopathological picture of SCAD in autopsy reports, although the relationship to the SCAD event is unclear.^10^ Inflammatory disorders and hereditary connective tissue disorders are reported in SCAD survivors, although precise mechanisms are not known.^11^

In this study, we aimed to investigate the spectrum of histological findings of SCAD from the largest reported autopsy series assembled to date and to study the dermal collagen ultrastructure of SCAD survivors to explore the implications of these findings both for clinical pathology and our understanding of the pathophysiology of this condition.

## 2. Methods

### 2.1 Study population

The study was conducted according to the principles of the Declaration of Helsinki. Autopsy-diagnosed SCAD cases who were referred with SCD were identified retrospectively from international cardiovascular pathology centres: the UK CRY (Cardiac Risk in the Young) database (St George’s hospital and Royal Brompton Hospital, London, UK); the Victorian Institute of Forensic Medicine (VIFM, Melbourne, Australia); and the University of Utrecht Medical Center (Utrecht, the Netherlands). Ethical approval for this study was granted in the UK (REC reference is 10/H0724/38), the VIFM [in accordance with section 2.3.5 of the NHMRC National Statement on Ethical Conduct in Research and the conditions set out in the Coroners Act 2008 (Victoria)—reference EC01/2017], and the Netherlands [the study and coding of human material met the criteria of the Netherlands Code of Conduct for the responsible use of human tissue for medical research as approved by the local Biobank Review Committee of the University Medical Center Utrecht (protocol number 15-252)]. Age- and sex-matched controls were identified from the CRY archives as individuals who suffered presumed sudden arrhythmic death syndrome (SADS) with a morphologically normal heart at autopsy, without evidence of SCAD or other obvious pathology. All cases underwent either coronial autopsy in accordance with a legal practice appropriate to the relevant national jurisdiction or routine autopsy for which consent was given by their next of kin. Permission for further evaluation of material from the autopsies without informed consent was provided by the relevant ethics committee as detailed above and in accordance with relevant national legislation for the handling of human tissue.

Consecutive SCAD cases and healthy volunteers (HV) were recruited to the UK Spontaneous Coronary Artery Dissection (UKSCAD) Study (ISRCTN42661582). Patients with SCAD are recruited from across the UK by self-referral, primary care physician referral, and referral from the clinical team at the index presenting hospital. HV were defined as individuals aged 18 and above who have never been diagnosed with any chronic condition and do not take any regular medications (except for hormonal contraception). All participants provided fully informed signed consent. The protocol was approved by the UK Health Research Authority (14/EM/0056).

**Figure 1:**
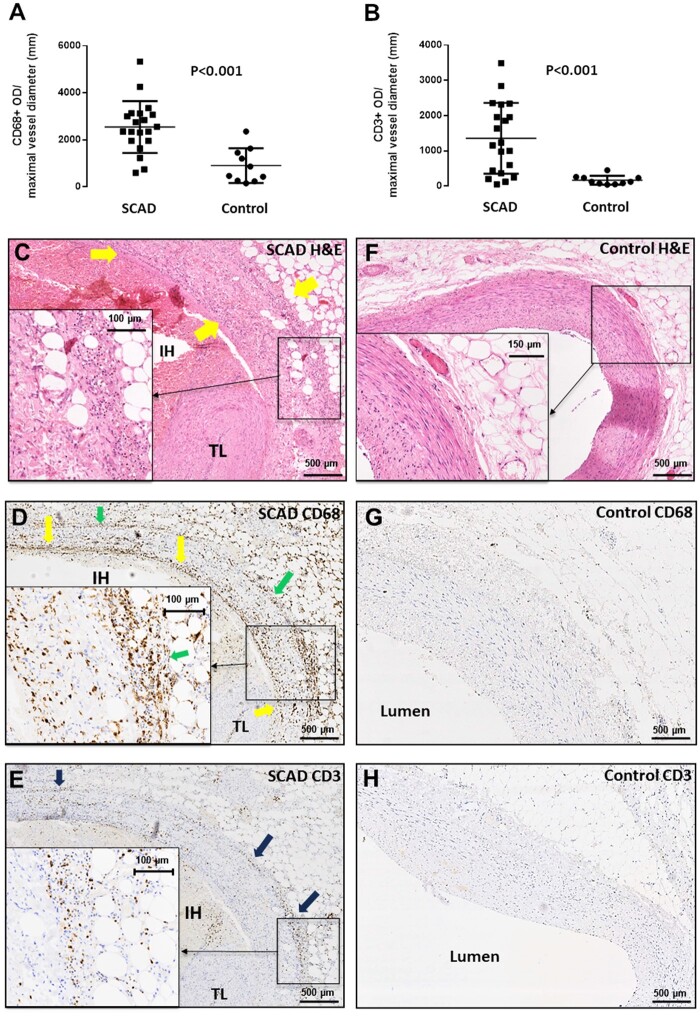
Composition of peri-adventitial inflammatory cell infiltrate in SCAD. Compared to age- and sex-matched control cases (*n* = 10), SCAD autopsy cases (*n* = 20) showed significantly higher infiltration with CD68+ macrophages (*A, P* < 0.001) and CD3+ T cells (*B, P* < 0.001). This infiltrate comprising lymphocytes, macrophages, and eosinophils was visualized in H&E staining (*C*) and spatially analysed with IHC. CD68+ cells were abundant throughout (*D*), whereas CD3+ T cells were less numerous and localized to the adventitial border (*E*). Control sections did not feature these findings (*F–H*). All comparisons between groups were made using unpaired *t*-test on log-transformed values.

A visual summary of the study design and different study populations/groups is shown in Supplementary material online, *FigureS1*.

### 2.2 Patient characteristics

For autopsy cases, demographics, circumstances of death, clinical data, and macroscopic findings for each case were obtained from the coroner referral letters and reports. Information was sought on previous cardiac signs/symptoms, history of pregnancy or post-partum at time of death, family history of connective tissue disorders or SCD, drug, and other medication use. *N* = 27 autopsy cases had sufficient details regarding the circumstances of death and prior symptoms described by the individual, based on which the time period between onset of symptoms and death could be determined as <24 h (defined as ‘rapid-onset death’) or ≥ 24 h (‘delayed-onset death’). For the SCAD cases and HV population, demographic information, medical history, and a detailed history of the SCAD event (if applicable) were obtained from medical records and directly from the subject. Pregnancy-associated SCAD (P-SCAD) was defined as SCAD occurring during gestation or within 12 months of delivery. Hypertension and dyslipidaemia were defined by the need for active treatment prior to SCAD.

#### 2.2.1 Histopathology and immunohistochemistry

Haematoxylin and Eosin (H&E), as well as Elastic Van-Gieson (EVG)-stained sections of culprit and non-culprit coronaries, were examined under light microscopy. All cases examined had at least one H&E-stained coronary artery section from the left main stem (LMS), as well as one of the proximal and one of the distal part of the three coronary arteries in addition to sections of the site of dissection.

Immunohistochemistry (IHC) was performed in a subset of *n* = 20 SCAD cases, where blocks of the SCAD-affected coronary artery were available. As a comparator control group, we randomly selected *N* = 10 age- and sex-matched SADS cases from the CRY archive. Sections were cut from paraffin-embedded tissue and fixed on SuperFrost slides. IHC was undertaken for the following targets: CD68 (MenaPath monoclonal KP1, Menarini Diagnostics, Berkshire, UK); CD3 (ab5690, Abcam, Cambridge, UK); CD31 (ab28364, Abcam), α-smooth muscle actin (ab7817, Abcam); CD34 (07-3403, ThermoFisher, UK). Staining was conducted on a Ventana Benchmark Ultra autostainer as per the manufacturer’s guidelines, using the Optiview DAB kit for detection of targets. To quantify VV density in the vasculature, IHC was undertaken for CD31 [Platelet endothelial cell adhesion molecule (PECAM), abundantly present in endothelial cell junctions]. VV was quantified by counting the number of CD31+stained vascular structures within the media and adventitia of the coronary sections. Adjustment for vessel size was performed by dividing the number of VV by the maximal diameter of the coronary section studied. Additional analyses were undertaken by quantifying the total CD31+ staining within the tunica media of the coronary.^12^ Staining for CD34, expressed on the surface of endothelial progenitor cells and mature endothelium, was used as a comparable positive control. All image analyses were performed using ImageJ software.^13^

#### 2.2.2 Electron microscopy

Dermal skin biopsies were collected, after local anaesthesia, through resection of a small ellipse (5–6 mm) on the inner side of the left upper arm. Skin biopsies were collected in 5 mL of cold filtered 2.5% glutaraldehyde solution (Glutaraldehyde 25% EM Grade Agar Scientific, Essex, UK) diluted in phosphate buffer (PB) 0.1 M (Sigma-Aldrich, UK). Each fixed tissue was then cut longitudinally into three smaller pieces of 1 mm width and further fixed in glutaraldehyde 2.5% solution from 2 h up to overnight. Tissue sections were then washed in PB 0.1 M and secondarily fixed in 1% osmium tetroxide (Agar Scientific, Essex, UK) dissolved in potassium ferricyanide 1.5% (Sigma-Aldrich, UK) for 90 min. Sections were then washed in distilled de-ionised water, dehydrated through a series of 70%, 90%, and 100% analytical grade ethanol (Sigma-Aldrich, UK), and processed in a gradually increasing concentrations of epoxy resin (Agar Scientific, Essex, UK) dissolved in propylene oxide (Sigma-Alrich, UK). Finally, the specimens were oriented in order to show the full thickness of the dermis and embedded in pure resin. Using a Leica UM UC7 microtome, the resin blocks were trimmed to thin sections (70 nm) and placed on copper grids (Agar Ltd, Essex, UK). Sections were counterstained using 2% uranyl acetate (Agar Scientific, Essex, UK), followed by the immersion in a lead citrate solution, an in-house solution obtained by mixing lead nitrate (Agar Ltd, Essex, UK) and sodium citrate (Sigma-Alrich, UK). The sections were viewed with a JEOL JEM-1400 transmission electron microscope (TEM) with an accelerating voltage of 100 kV using an Olympus Megaview III with iTEM software digital camera. Images of collagen fibrils, elastin, and fibroblast cells were all taken in the mid-reticular dermis. Collagen fibrils minimum diameter was measured in at least 5–6 images of transversal section orientation in at least three different collagen bundles. The measurements were performed by using a macro designed with the ImageJ software that allowed to measure about 2000–3000 fibril diameters per subject. Images of collagen fibrils in transverse sectional orientation were also examined for the presence of irregular fibrils (fibrils with irregular edges) in 8 to 10 different collagen bundles distributed in 4 to 5 areas of the copper grid. An average of 8 images of elastin and fibroblasts per subject were also taken and the widest diameter in each image, as well as for the diameters of irregular fibrils, was measured by post-analysis of the images with ImageJ. Elastin images were qualitatively analysed for the presence of features reported in hereditary connective tissue disorders such as frayed edges, moth-eaten edges, calcified microcavities, dense internal spots, thick surface coat, and indentations.^14,15^ A percentage was then calculated for each feature observed in each subject. All analysis was conducted blinded to SCAD and HV status.

### 2.3 Statistical analysis

Summary statistics are provided for all variables, including means ± standard errors for continuous variables and counts and percentages for categorical variables. Continuous variables were tested for normality using Kolmogorov–Smirnov test, and by visual inspection of the data.

When comparing the UKSCAD cohort with the autopsy cases, as well as in the histology and IHC experiment analyses, differences in continuous and categorical variables were tested using independent sample *t*-test or Mann–Whitney *U*-test and Fisher exact tests, respectively.

In the electron microscopy experiments, univariate and multivariable regression analyses were carried out to test the impact of the most relevant clinical data (group SCAD vs. HV; Beighton score >4 vs. <4; number of pregnancies 3+ vs. <3 and age) on the quantitative and qualitative variables measured in the collagen fibrils, elastin, and fibroblasts. For continuous outcomes linear regression was performed and mean differences presented. For count outcomes Poisson regression was employed and incident rate ratios presented. For binary outcomes, logistic regression was performed and odds ratios presented. All estimates are presented with 95% confidence intervals (CIs) and *P*-values. A *P*-value of <0.05 was considered statistically significant. All statistical analyses were performed using SPSS version 20.0 or GraphPad Prism version 8.0.

## 3. Results

### 3.1 Characteristics of autopsy cases and the UKSCAD cohort

A total of 36 autopsy cases of SCAD were studied from 1996 to 2016. None of the cases had a diagnosis of SCAD prior to death. The case series consisted of *n* = 29 females (81%) with an average age of 49.4 ± 2.5 (range 26–78 years). Three of the patients were P-SCAD, accounting for 10% of females in the case series. The prevalence of cardiovascular risk factors is shown in *Table 1*. None of the cases had a known inflammatory disorder, connective tissue disorder, or relevant family history.

**Table 1:** Demographics and cardiovascular risk factors of autopsy cases and UKSCAD Cohort

	Autopsy cases	UKSCAD cohort	*P*-value
	*n* = 36	*n* = 359	
Female, *n* (%)	29 (81%)	342 (95%)	0.0004
Post-partum,^a^*n* (%)	3 (10%)	30 (9%)	0.7223
Age (years)	49.4 ± 2.5	47.0 ± 0.5	0.3491
Body mass index (kg/m^2^)	29.6 ± 1.5	26.2 ± 0.3	0.0290
Active smoking, *n* (%)	5 (14%)	14 (4%)	0.0014
Hypertension, *n* (%)	7 (19%)	85 (24%)	0.6311
Dyslipidaemia, *n* (%)	7 (19%)	33 (9%)	0.0114
Diabetes mellitus, *n* (%)	1 (3%)	7 (2%)	0.4433

a Females only. Data presented as *N* (%) or mean ± standard error.

The demographics and risk factors were compared with the UKSCAD cohort (*Table 1*). Although in both groups most patients were female, there was a significantly higher percentage of female individuals in the UKSCAD cohort compared to the autopsy cases (342/359. 95% vs. 29/36, 81%; *P* = 0.0004). The percentage of P-SCAD cases was similar between the two groups. Autopsy cases had higher BMI (*P* = 0.0290), higher prevalence of active smoking (*P* = 0.0014), and dyslipidemia (*P* = 0.0114).

### 3.2 Macroscopic examination

**Figure 2:**
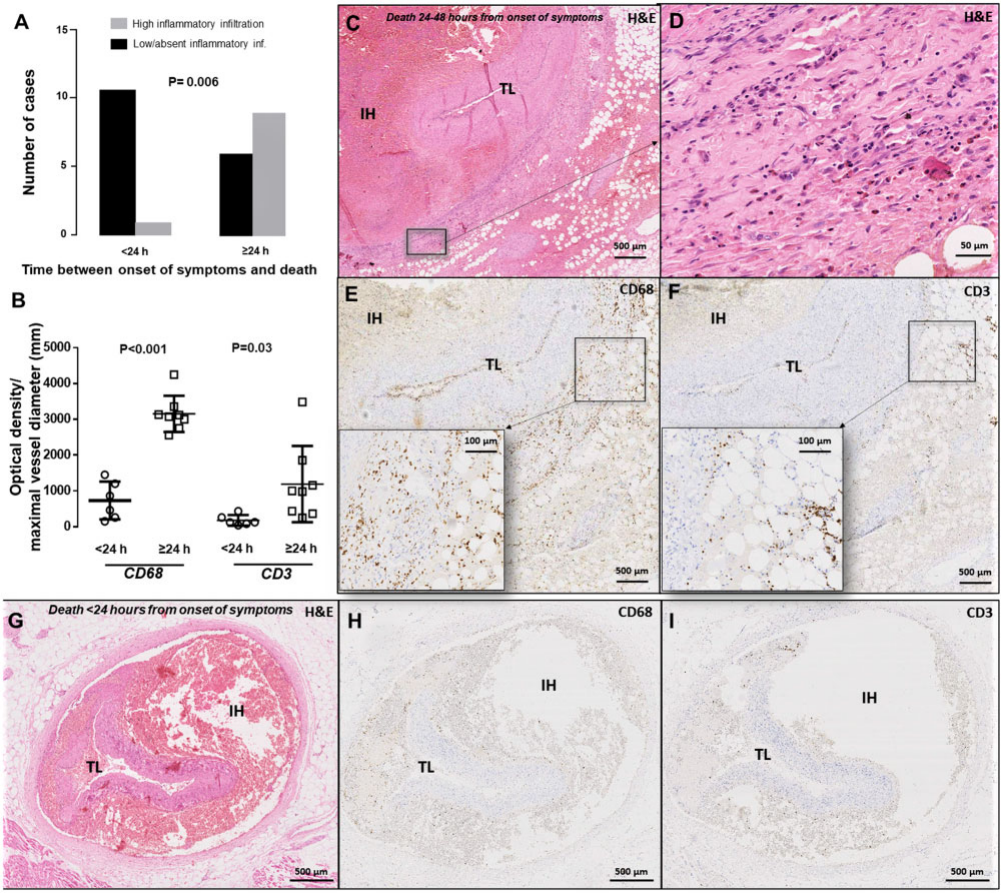
Association between the degree of peri-adventitial inflammatory infiltrate and time interval from SCAD symptom onset to death. Increased degree of peri-adventitial inflammatory cell infiltration was significantly associated with increased time from symptom onset to death (*A, n* = 27, *P* = 0.006, χ^2^ test). CD68+ and CD3+ staining showing a higher macrophage (*B*, CD68+, *P* < 0.001, *n* = 17), and T-cell infiltrate (*B*, CD3+, *P* = 0.03, *n* = 17) in cases with more than 24 h delay between onset of SCAD-related symptoms and death. Comparisons made using an unpaired *t*-test on log-transformed values. Panels (*C–I*) provide examples of autopsy cases belonging to the delayed-onset (*C–F*) vs. rapid-onset (*G–I*) death groups.

All autopsy cases involved a single coronary artery (example of typical macroscopic findings in Supplementary material online, *FigureS2*). Localization of SCAD in the autopsy series was compared with angiographic data from the SCAD-survivor cohort (*Table 2*). There was a significantly higher proportion of SCAD autopsy cases involving the LMS or proximal coronary artery segments compared to SCAD survivors (autopsy—20/36, 56% vs. survivors 64/359, 18%; *P* < 0.0001). These included *n* = 3 cases originating in the LMS but also extending into the left anterior descending (LAD) artery. There were significantly more SCAD cases involving the LAD, predominantly in the mid-distal vessel (survivors—221/359, 61% vs. autopsy cases—12/36, 33%; *P* = 0.003). In the autopsy case series, the extent of the dissection along the length of the culprit vessel varied from 5 to >50 mm.

**Table 2:** Anatomic localization of culprit lesions in autopsy cases and UKSCAD Cohort

	Autopsy cases (*n* = 36)	UKSCAD cohort (*n* = 359)	*P*-value
LMS (*n*, % total)	6 (17%)	16 (4%)	0.0095
LAD (*n*, % total)	14 (33%)	234 (65%)	0.0033
Proximal (*n*, % LAD)	9 (75%)	29 (13%)	0.001
Mid-distal (*n*, % LAD)	3 (25%)	192 (87%)	
LCx (*n*, % total)	6 (17%)	104 (29%)	0.1706
Proximal (*n*, % LCx)	2 (33%)	13 (14%)	
Mid-distal (*n*, % LCx)	4 (67%)	81 (86%)	
RCA (*n*, % total)	12 (33%)	68 (19%)	0.0501
Proximal (*n*, % RCA)	3 (25%)	6 (9%)	
Mid-distal (*n*, % RCA)	9 (75%)	61 (91%)	
Multi-vessel (*n*, % total)	0	33 (9%)	
Triple vessel (*n*, %)		6 (1.7%)	

LMS cases include cases where extension into the LAD was noted. LAD, LCx, and RCA cases include all cases where the origin of SCAD lesion was noted within the vessel, including multi-vessel cases.

LAD, left anterior descending artery; LCx, left circumflex artery; LMS, left main stem; RCA, right coronary artery.

In two-thirds of the autopsy cases (*n* = 24), there was no evidence of necrotic myocardium on either macro- or subsequent microscopic examination. Of the three P-SCAD cases in the autopsy series, two originated from the proximal LAD and one from the distal left circumflex (LCx), extending into the posterior descending artery and in contrast to non-P-SCAD autopsy cases, all P-SCAD cases were associated with necrosis of the underlying myocardium corresponding to the affected vessel.

### 3.3 Microscopic examination

The microscopy findings are presented systematically from adventitia to intima.

#### 3.3.1 Inflammatory cell infiltrate

There was substantial heterogeneity in the degree of inflammatory cell infiltrate in the sections studied. One-third (*n* = 12) of autopsy cases had minimal inflammatory cell infiltrate, whilst two-thirds of the cases (*n* = 24) displayed significant infiltration with neutrophils/macrophages, lymphocytes, and/or eosinophils in the adventitia with varying degrees of extension into the medial layer. *N* = 11 of those cases showed fully organized peri-adventitial fibrous tissue. There were only very rare giant cells around the elastic fibres of the dissected segments. In all cases, the inflammatory cell infiltrate was limited to sections containing the false lumen and was not present in healthy, non-dissected, proximal or distal sections of the culprit and non-culprit coronaries.

To compare the SCAD inflammatory cell infiltrate with the established histopathology of medium- and large-vessel arteritides, we performed IHC for CD68 (surface marker of macrophages) and CD3 (surface marker of T-lymphocytes) and compared with age- and sex-matched control cases. There was, as expected, significantly higher infiltration of CD68+ and CD3+ cells in the peri-adventitial tissues surrounding an SCAD section compared to control cases (*Figure 1A and B* and microphotographs *Figure 1C–H*). In SCAD cases with significant inflammation, there was abundant CD68+ staining throughout the adventitial infiltrate, extending into the perivascular adipose tissue and in the media surrounding the dissection plane and haematoma (*Figure 1D*). Similarly, there was significant, albeit less pronounced staining for CD3, which appeared to be more spatially localized over the adventitial border of the vascular wall, as well as the outer rim of the media and adventitial inflammatory infiltrate (*Figure 1E*).

We next assessed the association between time elapsed from symptom onset to death and degree of inflammatory infiltrate. Two researchers, both blinded to clinical details, independently analysed *n* = 27 cases for whom symptom-to-death time was available, semi-quantifying the degree of inflammatory cell infiltrate as ‘high’ or ‘low/absent’. There was 95% observer concordance. The degree of peri-adventitial inflammatory cell infiltration was significantly associated with a longer time period from symptom onset to death (*Figure 2A*, *P* = 0.006; e.g. *Figures 2C vs. 2G)*. Similarly, IHC staining for CD68+ (*Figure 2B, E & H*) and CD3+ (*Figure 2B, F & I*) showed a similar significant link between abundant cellular staining and longer time interval from symptom onset to death.

**Figure 3:**
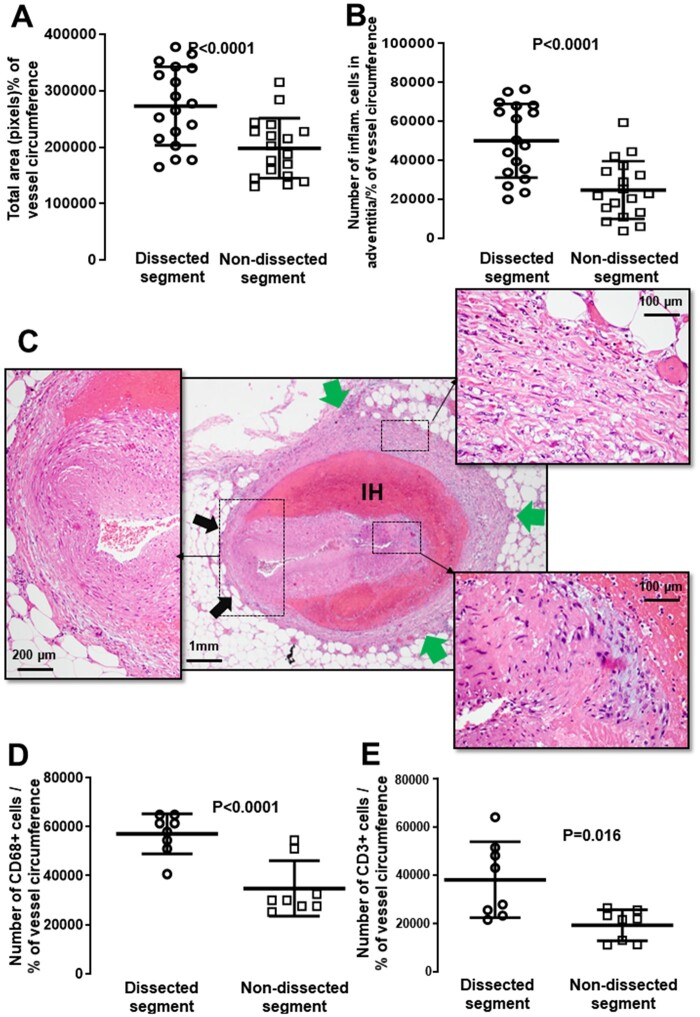
Association between the degree of peri-adventitial inflammatory infiltrate and proximity to dissected portion of the medial layer. In SCAD cases, we observed significantly higher inflammatory area (*A, P* < 0.0001; *n* = 18) and denser peri-adventitial inflammatory cell infiltrate (*B, P* < 0.0001, *n* = 18) adjacent to dissected segments vs. non-dissected coronary segments. In a typical SCAD section (*C*), there is denser reactive adventitial tissue (green arrows) surrounding the intramural haematoma (IH) vs. areas adjacent to healthy, non-dissected portions of the medial layer (black arrows). Similarly, IHC showed that the number of CD68+ macrophages (*D, P* < 0.0001, *n* = 8) and CD3+ T-lymphocytes (*E, P* = 0.016, *n* = 8) was higher in the adventitia surrounding the dissected vs. non-dissected coronary circumference. All comparisons between dissected and non-dissected segments were made using paired *t*-test.

In addition to this temporal association, we sought to establish a spatial association between inflammation and the dissected media. In the *n* = 18 autopsy cases that exhibited high inflammatory infiltrate, there was a significantly larger surface area of peri-adventitial reactive tissue adjacent to dissected vs. non-dissected segments of the coronary sections examined, after adjusting for the percentage of total vascular circumference affected (*Figure 3A*, *P* < 0.001). Furthermore, in H&E sections, the number of peri-adventitial inflammatory cells surrounding the dissected segment was significantly higher compared to the non-dissected segment, adjusted for the percentage of vessel circumference affected by the dissection (*Figure 3B*, *P* < 0.0001). These results were replicated when examining the number of CD68+ macrophages (*Figure 3D*, *P* < 0.0001) and CD3+ T-lymphocytes (*Figure 3E*, *P* = 0.016) corresponding to the dissected vs. non-dissected segment in IHC analysis.

#### 3.3.2 Vasa vasorum

**Figure 4:**
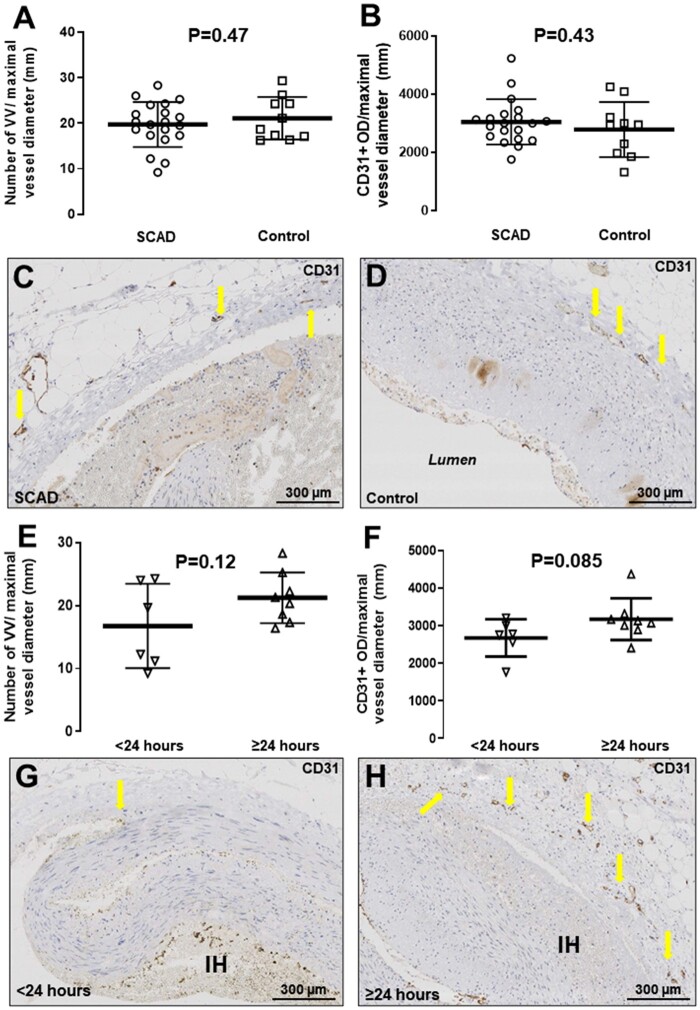
VV density in SCAD. VV in SCAD coronary sections (*n* = 20) vs. control cases (*n* = 10) (*A, P* = 0.47) or total CD31+ optical density in the vascular media adjusted for maximal vessel diameter (*B, P* = 0.43). Panels (*C* and *D*) are representative SCAD and control microphotographs of CD31-stained sections, respectively. VV density comparing rapid-onset (<24 h from symptom to death) to delayed-onset (>24 h) SCAD fatalities (*E* and *F, G* and *H* representative microphotographs for <24 (*n* = 6) and >24 h (*n* = 8) groups, respectively. *P* = NS). All comparisons between groups were made using an unpaired *t*-test on log-transformed values.

After adjusting for vessel diameter, no significant differences in the density of VV were found between SCAD sections and controls (*Figure 4A*). Total CD31 staining (PECAM-1, expressed on the surface of endothelial cells) in the media and adventitia also did not differ between the two groups (*Figure 4B–D*). When comparing SCAD autopsy cases with rapid- vs. delayed-onset death, we observed a trend towards denser VV (*Figure 4E*) and more abundant medial and adventitial CD31 staining (*Figure 4F–H*), although the association did not reach statistical significance. The distribution of CD34 staining was similar, confirming the structures stained as VV (Supplementary material online, *FigureS3*).

#### 3.3.3 Medial dissection and intramural haematoma

In most cases (*n* = 31, 86.1%), the dissection event occurred near the outer media, close to the adventitial border. In the remaining cases, the medial intramural haematoma was localized close to the internal elastic lamina (IEL).

**Figure 5:**
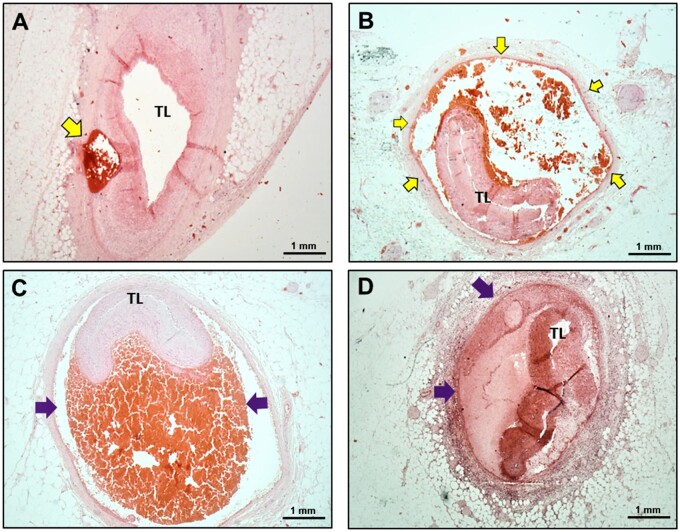
Intramural haematoma features from the SCAD autopsy case series. (*A* and *B*) Sequential H&E sections from the same autopsy case. Distally only a small proportion of the total vessel circumference is affected (*A*), leaving the true lumen (TL) relatively patent, whereas proximally the false lumen envelops almost the entire vessel causing significant luminal compression (*B*). (*C*) Intramural haematoma (purple arrow) displaying dense red clot with minimal fibrin formation. (*D*) Intramural haematoma (purple arrows) showing varying degrees of maturation with fibrin formation, with almost distinct ‘compartmentalization’ of segments within. Yellow arrows: intramural haematoma; TL, true lumen.

The proportion of the total coronary circumference affected varied widely both within and between cases. Some sections displayed a small intramural haematoma accounting for less than 10% of the vessel medial area (e.g. *Figure 5A*); on the other hand, more proximal sections belonging to the same case displayed a false lumen enveloping almost the entirety of the coronary circumference (*Figure 5B*).

The appearance and constituents of the false lumen were heterogeneous. Some cases displayed dense red clot with trapped white cell nuclei and minimal fibrin formation (*Figure 5C*). Other cases had varying degrees of mixed layers of fibrin formation, with almost distinct ‘compartmentalization’ of different segments of the intramural haematoma, giving a ‘trabeculated’ appearance, akin to the so-called lines of Zahn seen in fresh thrombus (*Figure 5D*).

#### 3.3.4 Intimal features and atherosclerotic changes

More than half (*n* = 28, 78%) of the autopsy cases studied displayed some intimal changes. These ranged from mild to moderate thickening (a recognized feature of ageing) but also included changes consistent with underlying atherosclerosis. Approximately half of the cases (*n* = 17, 47%) had mild to moderate atherosclerotic changes in both culprit and non-culprit coronary arteries. These changes were mostly limited to early neo-intima formation, with proliferation of vascular smooth muscle cells and sometimes the formation of foam cells in the intima. Only one case displayed significant atheroma (80% stenosis) on macroscopic examination, but in a non-dissected coronary vessel.

**Figure 6:**
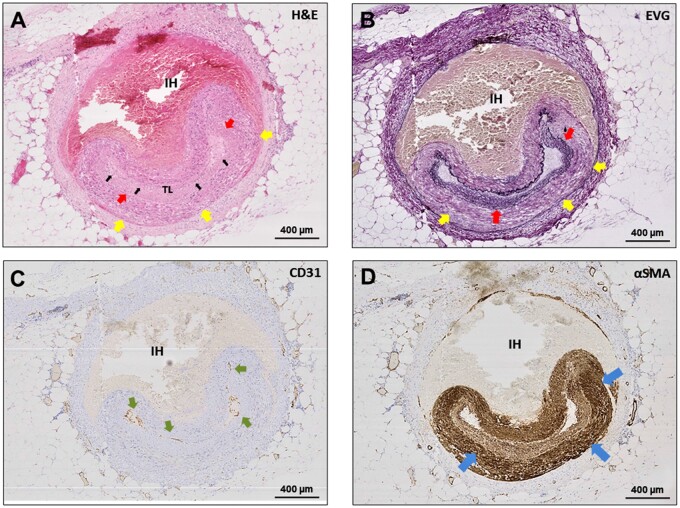
Intimal and medial layer features in SCAD. (*A*) H&E staining of SCAD lesion with medial dissection and intramural haematoma (IH) leading to compression true lumen (TL) compression (black arrows). Moderate fibro-elastic thickening of the endothelial layer (red arrows) but no abnormalities in structure or orientation of medial smooth muscle cells (yellow arrows). (*B*) EVG staining. Fibro-elastic intimal thickening is well-delineated (red arrows); the IEL clearly visualized and smooth muscle cell medial layer distinguished from intima and adventitia (yellow arrows). (*C*) CD31 staining shows mature endothelial cells on the intimal surface (green arrows). (*D*) αSMA showing normal pattern of staining in the non-dissected segment of the media (blue arrows).

No recognized histological features of fibromuscular dysplasia (FMD) were identified. FMD is characterized by thickening and proliferation of the intimal layer and obliteration of the medial layer through extensive, dense, deeply stained collagen deposition, as well as fragmentation of the internal and/or external elastic lamina. These features are prominent in the example provided in Supplementary material online, *FigureS4*, which displays two internal mammary arteries from our archives, showing typical FMD features. These features were absent in the SCAD autopsy cases studied: *Figure 6* shows a typical SCAD case, displaying a minor degree of intimal thickening and collagen deposition, with intact tunica media in the non-dissected segment and without fragmentation of the elastic laminae. Specifically, none of the cases examined demonstrated the extensive, dense, deeply stained collagen deposition seen in FMD (e.g. *Figure 6A and B*). To further confirm the presence of intact endothelial cells, we performed IHC staining for CD31 (PECAM-1). Staining for this mature endothelial cell marker showed the normal presence of endothelial cells in the intima of all histological sections studied (e.g. *Figure 6C*). EVG staining also did not reveal evidence of excessive collagen deposition in the intima or the media, which is distinct from the mild fibro-elastic intimal thickening seen in some cases (e.g. *Figure 6B*). In addition, IHC for α-smooth muscle actin showed a normal pattern of staining across the non-dissected segments of the media in SCAD sections (e.g. *Figure 6D*).

We did not observe evidence of IEL degradation or fragmentation in any of the sections studied or differences when compared to control cases (e.g. *Figure 6B*).

#### 3.3.5 Extra-coronary arterial findings

No FMD in extra-coronary arteries was reported on the autopsy reports of the SCAD cases. Complete non-coronary arterial material was, however, not available for examination. Non-coronary arteries were examined from 19 autopsy cases. No FMD was identified from 12 renal arteries, 7 renal arterioles, 1 splenic artery, 1 vertebral artery, 1 aorta, and 1 cerebral artery examined.

### 3.4 Electron microscopy

**Figure 7:**
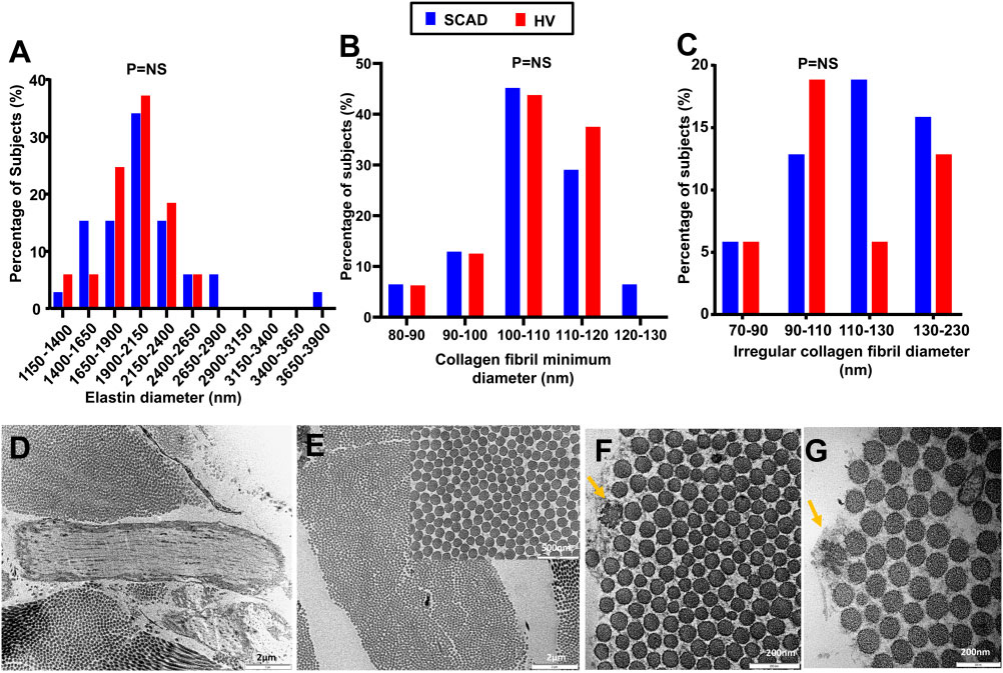
Ultrastructural analysis of the main ECM components in SCAD patients vs. HV. Elastin diameter (*A*), collagen fibril minimum diameter (*B*) and irregular collagen fibril diameter (*C*) distributions in SCAD (*n* = 31) vs. HV (*n* = 16). Representative images of elastin (*D*), collagen fibrils (*E*), and irregular fibrils (*F* and *G*). *T*-test between the averages, standard deviations, minimum and maximum values, and ranges of these parameters was performed between SCAD and HV. No significant differences were observed.

Dermal connective tissue from 31 patients and 16 HV was assessed by trasmission electron microscopy . Demographic characteristics and cardiovascular risk factors can be found in *Table 3*. No significant differences were found in the size of the major constituents of extracellular matrix (*Figure 7*); fibroblasts and their subcellular synthetic organelles (Supplementary material online, *FigureS5*); or features of elastin damage (Supplementary material online, *FigureS6*) between SCAD cases and HV.

**Table 3:** Demographics, cardiovascular risk factors, and SCAD event details of SCAD cases and HV recruited in the electron microscopy studies

	SCAD cases (*n* = 31)	HV (*n* = 16)
Age at biopsy (years)	45.8 ± 1.34	44.0 ± 1.49
Body mass index (kg/m^2^)	27.15 ± 1.13	26.37 ± 01.82
Active smoking, *n* (%)	1 (3.1%)	0
Hypertension, *n* (%)	8 (25%)	0
Dyslipidaemia, *n* (%)	1 (3.1%)	0
Diabetes mellitus, *n* (%)	0	0
P-SCAD, *n* (%)	5 (15.6%)	N/A
Age at SCAD event (years)	42.4 ± 1.39	N/A
Multi-vessel SCAD, *n* (%)	5 (15.6%)	N/A
Recurrent SCAD, *n* (%)	4 (12.5%)	N/A

Continuous variable values presented as mean ± SEM.

P-SCAD, pregnancy-associated SCAD; SCAD, spontaneous coronary artery dissection.

Univariate and multivariable analyses are presented in Supplementary material online, *TablesS1* and *S2*. This demonstrated a significant effect of age on minimum collagen fibril diameter (*P* = 0.0011), the number of irregular fibrils (*P* = 0.015), and elastin calcified microcavities (*P* = 0.0031). The number of elastic calcified microcavities was also significantly different (*p* = 0.0046) between those with low and high beighton score. In addition, significant differences between SCAD and HV were found for elastin thick surface coat (*P* = 0.0285) and elastin calcified microcavities (*P* = 0.0491).

## 4. Discussion

We present the largest study to date of SCAD coronary histopathology and the first systematic assessment of dermal collagen ultrastructure in SCAD survivors. We report, firstly, that myocardial necrosis is absent in a majority of autopsy cases suggesting a rapid or arrhythmic death. Secondly, inflammatory infiltration develops over time and likely constitutes a healing response to injury. Thirdly, we find no evidence of endothelial/intimal injury, no coronary histological features of FMD, and no evidence of an increased VV density in SCAD. Finally, we show no ultrastructural differences in dermal collagen and no evidence of changes in the cellular activity of skin fibroblasts in SCAD. Nevertheless, some features of elastin damage do appear to significantly differ between the two groups.

The study findings have important implication for the autopsy assessment of SCD. The presence of more proximal dissections when compared to patients surviving to angiography is consistent with higher-risk cases leading to fatality. However, the lack of myocardial infarction in a majority of cases suggests many deaths are arrhythmic and an absence of myocardial necrosis cannot rule out this diagnosis. These findings also suggest some patients with shorter, more distal SCAD presenting with arrhythmic death may be missed at autopsy unless this diagnosis is carefully excluded by systematic assessment of the entire coronary tree, as suggested by a previous smaller case series.^3^ The finding that P-SCAD was associated with more extensive myocardial infarction requires validation but is consistent with a number of studies reporting P-SCAD is a more extreme phenotype.^16,17^ This, coupled with the recent demonstration that post-menopausal women with SCAD may have a more benign phenotype than pre-menopausal women,^18^ suggests changes in female sex hormones may have a role in determining the severity of SCAD at presentation, although the mechanism remains unclear.

A number of hypotheses as to the arterial vulnerability and pathophysiological mechanism underlying SCAD have been proposed, to which our data provide novel insights. Although it was not possible to serially section the entire coronary tree of affected patients, no structural abnormalities of the coronary intima or the IEL were demonstrated, as might be expected for a spontaneous tear to develop (as implied from the inside-out hypothesis). Intracoronary imaging has provided evidence of false lumen pressurization prior to the development of fenestrations between false and true lumens and angiographic findings have shown these fenestrations occur after the development of intramural haematoma and not as a pre-requisite for SCAD.^6,19^ These findings taken together are supportive of the outside-in hypothesis as the predominant mechanism for SCAD. Additionally, coronary microvessels have been proposed as the potential source of intramural bleeding in SCAD. A previous intracoronary imaging study reported an increase in VV density in SCAD coronaries^5^ although this was not confirmed in a subsequent larger series.^6^ This histological study confirms no evidence for increased VV density suggesting that absolute vessel density may be less important than the vulnerability of traversing microvessels and the disrupting forces to which they are subjected. It has been demonstrated that adventitial vasoactive factors play an important role in regulating coronary arterial tone leading to speculation of an additional potential element to the outside-in pathophysiological hypothesis of SCAD.^20^

A significant proportion of patients with SCAD have been shown to have co-existent remote arteriopathies, particularly the ‘string-of-beads’ sign of radiological FMD.^1,2^ The exact proportion of SCAD cases with extra-coronary arteriopathies is unclear due to variations in the definitions and imaging modalities used in different studies.^1,21^ One recent study even reported 100% of SCAD cases had radiological arterial ‘abnormalities’ of some sort.^22^ This has led to speculation that SCAD arises primarily as a complication of pre-existent coronary histological FMD.^23^ In this study, the typical histopathological features of FMD were not seen in the coronary artery sections studied, suggesting that changes of localized coronary histological FMD are not a pre-requisite to SCAD in many cases. It is therefore likely that other, more subtle changes in the coronary vessel wall, such as differences in cell–cell adhesion or extracellular matrix function, are responsible for the vulnerability to SCAD. Histological FMD was not found on a limited review of available non-coronary arterial material from the autopsy cases. This may represent incomplete sampling but it remains to be confirmed that the ‘string-of-beads’ appearance of radiological FMD seen in SCAD invariably corresponds to histological changes consistent with the pathological definitions of FMD. The only reported post-mortem case presenting the gross pathological appearances of a renal artery ‘string-of-beads’, does not describe the histological findings of this artery.^24^ In this study, we are unable to definitively address the question as to whether the coronary histology of patients with SCAD and extra-coronary arteriopathies (including the radiological string-of-beads) differs from SCAD cases without such arteriopathies. A future prospective series with systematic serial sectioning of relevant arterial beds will be required to address these questions definitively. The low rates of atherosclerotic changes seen in the autopsy cases may reflect the low-risk profile of this predominantly female population but are also consistent with recent findings suggesting an opposing influence of common genetic variants on SCAD vs. ischaemic heart disease risk.^25,26^

Previous histopathological case reports have described SCAD as a mono-arteritis because of the density of the reported associated inflammatory infiltrate.^7,8^ Our study presents the most comprehensive evidence so far supportive that coronary inflammation in SCAD is a time dependent and localized healing response to the injury rather than a causal vasculitic process. This inflammatory infiltrate is distinct from that of medium- and large-vessel arteritides: There were scarcely any giant cells noted, a predominant feature in giant cell arteritis (GCA).^27^ The predominant cell type was CD68+ macrophages, as opposed to GCA and Takayasu’s arteritis, where the infiltrate is primarily CD3+ T-cell abundant.^27^ Eosinophilic infiltration was not a consistent global feature across the SCAD case series as described in eosinophilic coronary periarteritis.^28^ Our findings are consistent with the fact that, although inflammatory disorders are often reported as a predisposing condition in SCAD,^11^ rates of inflammatory diseases are probably similar to the general population.^29,30^

SCAD is associated with hereditary connective tissue disorders in a small proportion of cases.^9,31,32^ Features of hypermobility have also been described in a subgroup of SCAD survivors. This has led to speculation that even without a monogenetic cause, abnormalities of connective tissue might be a common mechanism underlying SCAD.^1,2^ Abnormalities of dermal collagen ultrastructure have been shown in a range of established connective tissue disorders.^15^ We found no generalizable difference in a range of connective tissue ultrastructural features on blinded analysis. Importantly, previously reported effects of age^33^ on collagen fibril size and the number of irregular fibrils were confirmed, providing effectively a positive control within the analysis. Some features of elastin damage were different between HV and SCAD survivors, suggesting a possible underlying predisposition towards more unstable elastin in these patients. These findings are hypothesis-generating and will require further validation but are in keeping with recent genetic studies^9,34^ showing causal connective tissue disorder variants in SCAD affect only <5% of index cases and suggests ultrastructural changes in dermal connective tissue are not a common feature in SCAD. Future assessment in demographic and genetic subgroups will be of interest.

### 4.1 Limitations

SCAD leading to SCD is rare, thus making a prospective unbiased design logistically impossible. As a retrospective observational study, we cannot conclude that the associations demonstrated are causative. All SCAD autopsy cases were initially referred for clinical autopsy and as such, it was impossible to employ a uniform methodology or sequential sectioning of the entire length of the coronary tree. Our ethical permissions did not permit genetic analysis of autopsy cases. As most autopsies are initially performed by non-cardiovascular pathologists and the heart (only) is retained for later examination by a cardiovascular pathologist, limited non-coronary arteries were available for assessment. Interpretation of the frequency of non-coronary arteriopathies is therefore limited by incomplete sampling. The numbers of included patients will impact on power meaning small effects in the measured indices may not be demonstrated. Dermal connective ultrastructure was used as a surrogate for coronary connective tissue as trasmission electron microscopy could not be undertaken on the dissected coronaries, given that fresh tissue is required.

## 5. Conclusions

Care is required during autopsy for SCD to exclude SCAD, particularly when affecting more distal coronary locations and presenting without myocardial necrosis. This study found no supporting evidence for a causal role for peri-coronary inflammation, which is more likely an evolving response to injury than a coronary mono-arteritis triggering an SCAD event. We also found no evidence to support the inside-out hypothesis, no features of coronary FMD, and no evidence for an increased VV density as the primary source for intramural bleeding. Finally, we find no generalized changes in dermal collagen ultrastructure, suggesting these changes may not be a prime pathophysiological driver in most patients.

## Supplementary material

Supplementary material is available at *Cardiovascular Research* online.

## Authors’ contribution

All authors contributed to the design of the work, interpretation of the data and the final writing of the manuscript. M.M., F.S., and A.A.B.-C. additionally contributed to the acquisition and analysis of the data and C.B. to the statistical analysis of the data. D.A. and M.N.S. are accountable for all aspects of the work and undertake to ensure that questions related to the accuracy or integrity of any part of the work are appropriately investigated and resolved.

## Supplementary Material

cvab183_Supplementary_Data

## Data Availability

The data underlying this article will be shared on reasonable request to the corresponding author.
