# Erratum to: Vascular histopathology and connective tissue ultrastructure in spontaneous coronary artery dissection: pathophysiological and clinical implications

**DOI:** 10.1093/cvr/cvac070

**Published:** 2022-05-12

**Authors:** Marios Margaritis, Francesca Saini, Ania A Baranowska-Clarke, Sarah Parsons, Aryan Vink, Charley Budgeon, Natalie Allcock, Bart E Wagner, Nilesh J Samani, Jan von der Thüsen, Jan Lukas Robertus, Mary N Sheppard, David Adlam

Cardiovascular Research, 2021, https://doi.org/10.1093/cvr/cvab183

In the originally published version of this manuscript, the primary affiliation for Jan Lukas Robertus was omitted in error. Jan Lukas Robertus' primary affiliation is ‘Department of Pathology, Royal Brompton Hospital, London, SW3 6NP, UK'. This error has been corrected. The publisher apologizes for the error.

