# Supplementary material for: Vascular histopathology and connective tissue ultrastructure in spontaneous coronary artery dissection: pathophysiological and clinical implications
Source: Cardiovasc Res. 2021 May 28;118(7):1835–48. doi: 10.1093/cvr/cvab183 (PMC9215198; doi:10.1093/cvr/cvab183)
Supplement: cvab183_Supplementary_Data [file cvab183_supplementary_data.pdf]

Supplementary Tables & Figures

Vascular Histopathology and Connective Tissue Ultrastructure in Spontaneous Coronary Artery Dissection: Pathophysiological and Clinical Implications

Marios Margaritis MRCP DPhil[*1], Francesca Saini PhD[*1], Ania Baranowska-Clarke MRes[1], Sarah Parsons MBBS[2], Aryan Vink MD PhD[3], Charley Budgeon PhD[1,4], Natalie Allcock[1], Bart E Wagner BSc[5], Nilesh J. Samani MD FRCP[1], Jan von der Thüsen MD PhD[6] , Jan Lukas Robertus MD PhD[7], Mary N Sheppard MD[#8], David Adlam DPhil FRCP[#1]

[1]Department of Cardiovascular Sciences and National Institute for Health Research Leicester Biomedical Research Centre, Glenfield Hospital, Groby Road, Leicester LE3 9QP, UK
[2]Department of Forensic Medicine, Victorian Institute of Forensic Medicine, Monash University, Melbourne, VIC, Australia
[3]Department of Pathology, University Medical Centre Utrecht, Utrecht, The Netherlands
[4]School of Population and Global Health, University of Western Australia, Perth, WA 6009, Australia; Core Biotechnology Services, College of Life Sciences, University of Leicester, LE1 7JA, UK
[5]Electron Microscopy, Histopathology Department, Royal Hallamshire Hospital, Sheffield Teaching Hospitals, Sheffield, S10 2JF, UK
[6]Department of Pathology, Erasmus MC, University Medical Centre Rotterdam, Rotterdam, The Netherlands, PO Box 2040, 3000 CA
[7]Department of Pathology, Royal Brompton and Harefield Hospitals, Guy's and St Thomas' NHS Foundation Trust, SW3 6NP, UK; National Heart & Lung Institute, Imperial College London, London, SW3 6LY, UK
[8]CRY Department of Cardiovascular Pathology, Molecular and Clinical Sciences Research Institute, St Georges Medical School, London SW17 0RE, UK

* The first two authors contributed equally to the study
[#] Joint senior authors

Corresponding authors.
Tel: +441162044751; fax: +441162875792, E-mail: da134@le.ac.uk (D.A.); Tel:+442087255112; fax +442087255139, E-mail: msheppar@sgul.ac.uk (M.S.)

Supplementary table 1– Transmission Electron Microscopy quantitative analyses & association with study subject demographics/risk factors

	Category	Univariate			Multivariate		
		Coefficient	95% CI	P-value	Coefficient	95% CI	P-value
**Mean of collagen min ferret diameters (length)**							
Intercept		-	-	-	133.38	(116.12, 150.64)	**<0.0001**
Age	1 year increase	-0.58	(-0.94, -0.22)	0.0022	-0.65	(-1.02, -0.28)	0.0011
Pregnancy	3+ vs <3	2.73	(-2.5, 7.95)	0.2984	2.78	(-2.27, 7.83)	0.2726
Group	SCAD vs HV	0.70	(-4.83, 6.23)	0.8008	3.15	(-2.45, 8.75)	0.2626
Beighton Score	>4 vs <4	-0.87	(-6.4, 4.65)	0.7519	-3.02	(-8.93, 2.89)	0.3083
**Elastin Diameters**							
Intercept		-	-	-	1574.49	(622.59, 2526.38)	**0.0018**
Age	1 year increase	7.22	(-12.52, 26.95)	0.4654	5.76	(-14.64, 26.16)	0.5716
Pregnancy	3+ vs <3	208.21	(-44.5, 460.92)	0.1040	229.38	(-44.15, 502.91)	0.0980
Group	SCAD vs HV	78.27	(-194.86, 351.39)	0.5667	133.93	(-171.7, 439.55)	0.3815
Beighton Score	>4 vs <4	-52.03	(-325.72, 221.66)	0.7036	-16.03	(-335.72, 303.66)	0.9199
**Fibroblast diameters**							
Intercept		-	-	-	3157.75	(1882.8, 4432.71)	**<0.0001**
Age	1 year increase	-4.66	(-30.62, 21.31)	0.7196	-0.07	(-27.39, 27.25)	0.9957
Pregnancy	3+ vs <3	-92.17	(-431.95, 247.6)	0.5875	-58.82	(-425.19, 307.55)	0.7475
Group	SCAD vs HV	-69.22	(-427.6, 289.15)	0.6991	-185.15	(-594.5, 224.2)	0.3666
Beighton Score	>4 vs <4	184.10	(-170.6, 538.79)	0.3014	242.34	(-185.84, 670.53)	0.2598
**Irregular fibrils diameters**							
Intercept		-	-	-	163.18	(37.66, 288.7)	0.0136
Age	1 year increase	-1.01	(-3.14, 1.12)	0.3366	-0.99	(-3.58, 1.61)	0.4371
Pregnancy	3+ vs <3	-2.67	(-30.14, 24.8)	0.8422	-1.89	(-36.25, 32.46)	0.9094
Group	SCAD vs HV	0.22	(-29.92, 30.36)	0.9881	0.03	(-34.2, 34.26)	0.9985
Beighton Score	>4 vs <4	6.60	(-21.55, 34.75)	0.6315	0.61	(-39.33, 40.55)	0.9749

**Percentage of Irregular fibrils**							
Intercept		-	-	-	45.82	(-6.44, 98.08)	0.0822
Age	1 year increase	-0.59	(-1.54, 0.35)	0.2078	-0.52	(-1.6, 0.56)	0.3264
Pregnancy	3+ vs <3	-9.19	(-20.91, 2.53)	0.1181	-7.46	(-21.76, 6.85)	0.2887
Group	SCAD vs HV	5.03	(-8.39, 18.45)	0.4451	2.85	(-11.41, 17.1)	0.6804
Beighton Score	>4 vs <4	9.16	(-2.95, 21.27)	0.1311	1.87	(-14.76, 18.5)	0.8164
**Number of irregular fibrils^**							
Age	1 year increase	0.94	(0.9, 0.99)	0.0109	0.95	(0.9, 0.99)	**0.0150**
Pregnancy	3+ vs <3	0.81	(0.45, 1.44)	0.4667	0.96	(0.53, 1.76)	0.9011
Group	SCAD vs HV	1.86	(0.92, 3.74)	0.0831	1.83	(0.83, 4.03)	0.1343
Beighton Score	>4 vs <4	1.63	(0.91, 2.91)	0.0999	1.06	(0.53, 2.1)	0.8717

^Poisson regression with incident rate ratios (and 95% CI) presented.

Supplementary table 2 - Qualitative Transmission Electron Microscopy data & association with study subject demographics/risk factors

	Category	Univariate			Multivariate		
		Coefficient	95% CI	P-value	Coefficient	95% CI	P-value
**Elastin frayed edges#**							
Intercept		-	-	-	30.65	(-21.54, 82.84)	0.2425
Age	1 year increase	0.41	(-0.7, 1.51)	0.4615	0.19	(-0.93, 1.31)	0.7375
Pregnancy	3+ vs <3	12.83	(-1.21, 26.87)	0.0724	9.77	(-5.23, 24.76)	0.1959
Group	SCAD vs HV	-4.03	(-19.32, 11.25)	0.5977	3.26	(-13.49, 20.02)	0.6963
Beighton Score	>4 vs <4	-14.65	(-29.34, 0.04)	0.0506	-12.25	(-29.78, 5.28)	0.17
**Elastin thick surface coat**							
Intercept		-	-	-	-1.20	(-71.44, 69.04)	0.9727
Age	1 year increase	0.90	(-0.6, 2.41)	0.2318	0.61	(-0.89, 2.12)	0.4149
Pregnancy	3+ vs <3	10.37	(-9.39, 30.13)	0.296	12.70	(-7.48, 32.88)	0.2111
Group	SCAD vs HV	18.98	(-1.3, 39.26)	0.0658	25.36	(2.8, 47.91)	**0.0285**
Beighton Score	>4 vs <4	-4.59	(-25.61, 16.43)	0.6622	-9.33	(-32.92, 14.26)	0.4292
**Elastin dense internal spots**							
Intercept		-	-	-	2.64	(-53.81, 59.09)	0.9252
Age	1 year increase	1.13	(-0.04, 2.29)	0.0577	1.18	(-0.03, 2.39)	0.0563
Pregnancy	3+ vs <3	12.02	(-3.48, 27.52)	0.1254	13.87	(-2.35, 30.09)	0.0918
Group	SCAD vs HV	2.56	(-14.19, 19.3)	0.76	2.06	(-16.06, 20.19)	0.8194
Beighton Score	>4 vs <4	-0.63	(-17.39, 16.13)	0.94	6.41	(-12.55, 25.37)	0.499
**Elastin indentations#**							
Intercept		-	-	-	27.70	(-34.34, 89.73)	0.3727
Age	1 year increase	0.53	(-0.79, 1.84)	0.4228	0.35	(-0.98, 1.68)	0.6014
Pregnancy	3+ vs <3	18.03	(1.54, 34.53)	0.0328	17.83	(0, 35.65)	**0.05**
Group#	SCAD vs HV	2.19	(-16.08, 20.46)	0.8104	8.79	(-11.13, 28.7)	0.3784
Beighton Score	>4 vs <4	-9.62	(-27.67, 8.43)	0.2888	-6.42	(-27.25, 14.41)	0.5373

**Elastin calcified microcavities***							
Age	1 year increase	0.89	(0.8, 0.99)	0.0267	0.80	(0.69, 0.93)	**0.0031**
Pregnancy	3+ vs <3	2.63	(0.81, 8.55)	0.1093	2.61	(0.56, 12.28)	0.2246
Group	SCAD vs HV	0.94	(0.28, 3.13)	0.9165	7.49	(1.01, 55.71)	**0.0491**
Beighton Score	>4 vs <4	0.21	(0.06, 0.81)	0.0229	0.03	(0, 0.33)	0.0046
**Moth eaten***							
Age	1 year increase	1.04	(0.95, 1.13)	0.453	1.03	(0.94, 1.14)	0.5045
Pregnancy	3+ vs <3	2.23	(0.69, 7.21)	0.1813	2.44	(0.69, 8.65)	0.1671
Group	SCAD vs HV	1.21	(0.36, 4.06)	0.7628	1.43	(0.35, 5.85)	0.6158
Beighton Score	>4 vs <4	0.83	(0.25, 2.79)	0.7628	1.05	(0.24, 4.54)	0.9513

*Logistic regression with odds ratios (and 95% CI) presented

As part of a whole exome sequencing study including all but one of the SCAD patients, 1 patient was identified with a truncating variant in COL3A1 (c.712C>T, p.Arg238. For the characteristics presented, this patient is not an outlier. In the measure of elastin with frayed edges and indentations only 4 SCAD cases recorded higher values. However, elastin fibres were not further degenerated into moth eaten edges and no thick surface coat and dense internal spots were observed. Fibroblast cell activity was present but low in this case and no autophagy was observed.

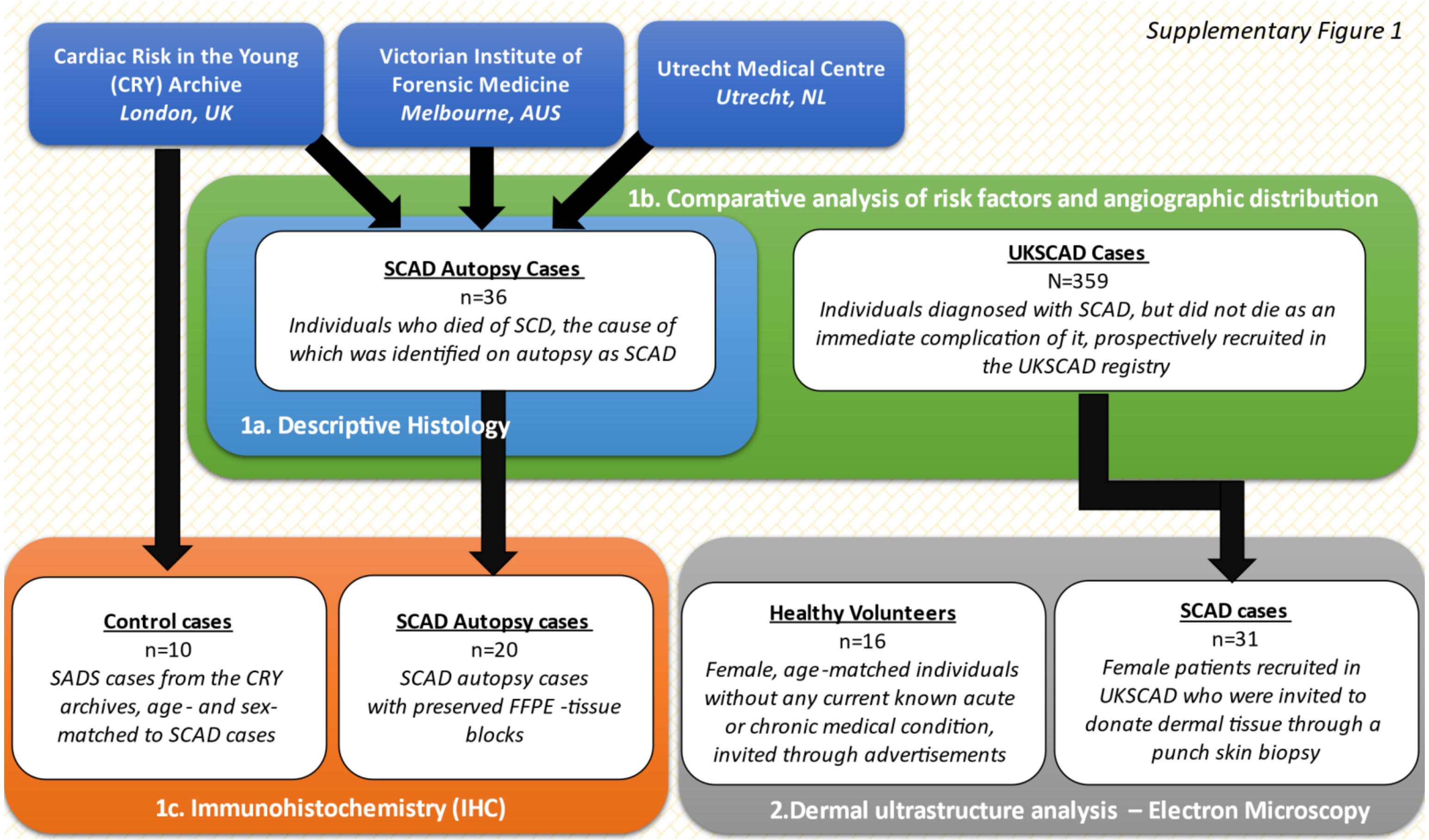

Supplementary Figure S1. Study design and group populations. Through an international collaboration of three pathology centres, n=36 sudden cardiac death autopsy cases were identified, where the cause of death was determined post-mortem to have been SCAD. The histopathological picture was described in the first part of study 1. In the second part, the demographics and risk factors of these autopsy cases were compared to n=359 survivors of SCAD recruited in the UKSCAD registry. In the third part, a subset of n=20 of the original n=36 autopsy SCAD cases was immune-stained for various targets and compared against n=10 age- and sex-matched control cases who suffered sudden arrhythmic death syndrome (SADS) and had a morphologically normal heart on autopsy. In study 2, n=16 age-matched healthy female volunteers and n=31 female SCAD cases (approached through UKSCAD to voluntarily participate after written, informed consent was obtained) underwent an elliptical skin biopsy; dermal fibroblasts were isolated and studied using Transmission Electron Microscopy.

Supplementary Figure 2

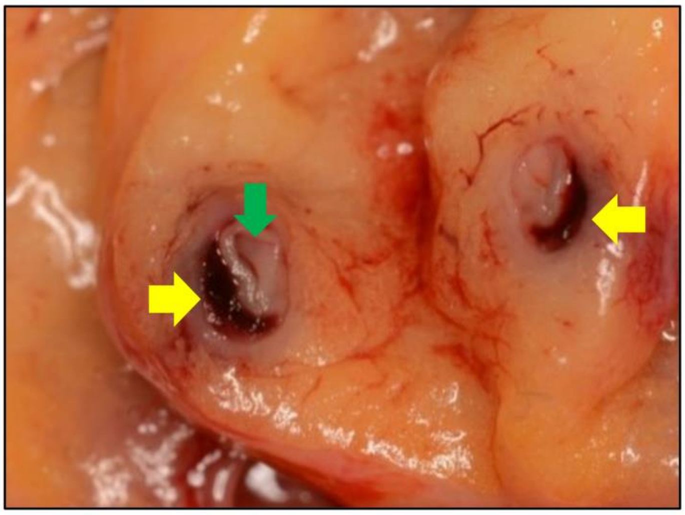

Supplementary Figure S2. Macroscopic appearance of SCAD on autopsy. A section of a right coronary artery shows presence of a dissection plane with intramural haematoma in the media (yellow arrows), whereas the true lumen is compressed (green arrow).

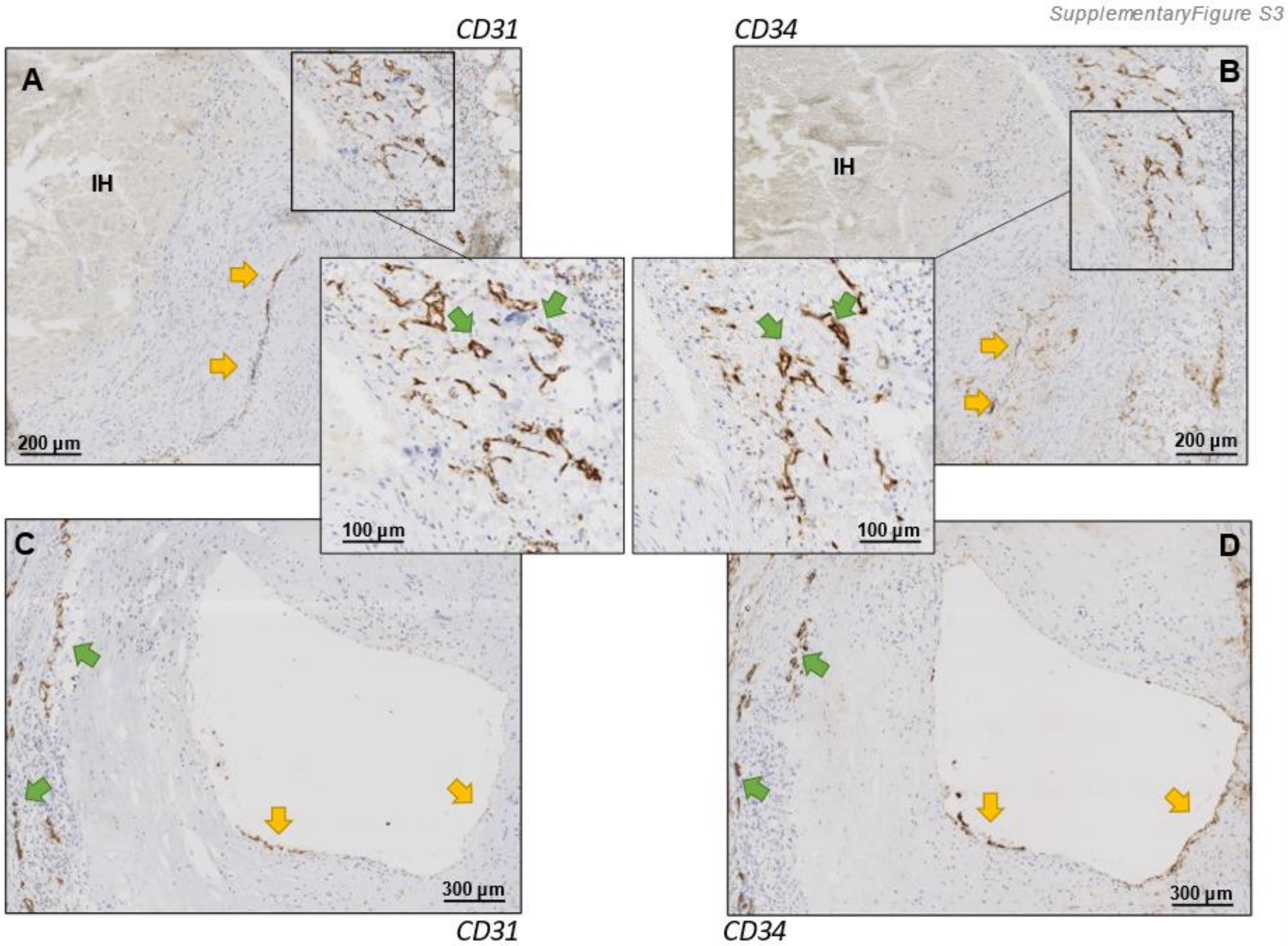

Supplementary Figure S3 Visualisation of vasa vasorum via CD31 and CD34 immunostaining. Staining for CD31 selectively highlights endothelial cells, visualising both the endothelial cell layer of the intima (yellow arrows), as well as the vasa vasorum of the media (green arrows). Near-identical pattern of staining was obtained after staining for CD34, which is also expressed in endothelial progenitor cells and in mature epithelial cells. Panels A & B are representative examples of sequential cuts from the same artery of a SCAD autopsy case, stained for CD31 and CD34, respectively. Panels C&D are representative examples of a control autopsy case.

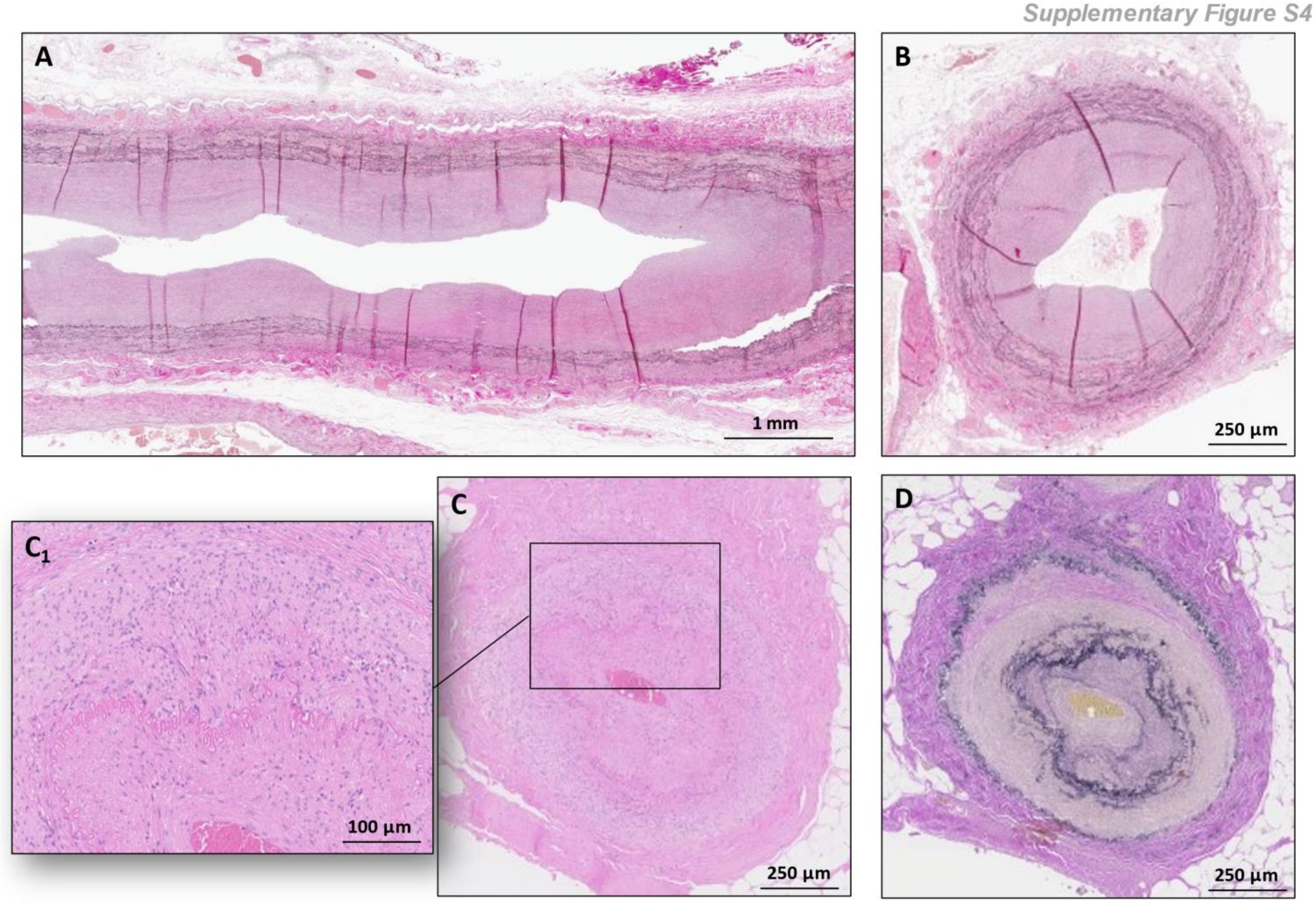

Supplementary Figure S4. Exemplary features of fibromuscular dysplasia in sections of two internal mammary arteries from the Utrecht Medical Centre archive. Top panels: Longitudinal section (A) and transverse section (B) of an internal mammary artery demonstrating features of fibromuscular dysplasia (FMD), stained with Elastic Van-Giesson (EVG). **Bottom panels:** Haematoxylin & Eosin (C) and EVG staining (D) of a second internal mammary artery displaying features of FMD.

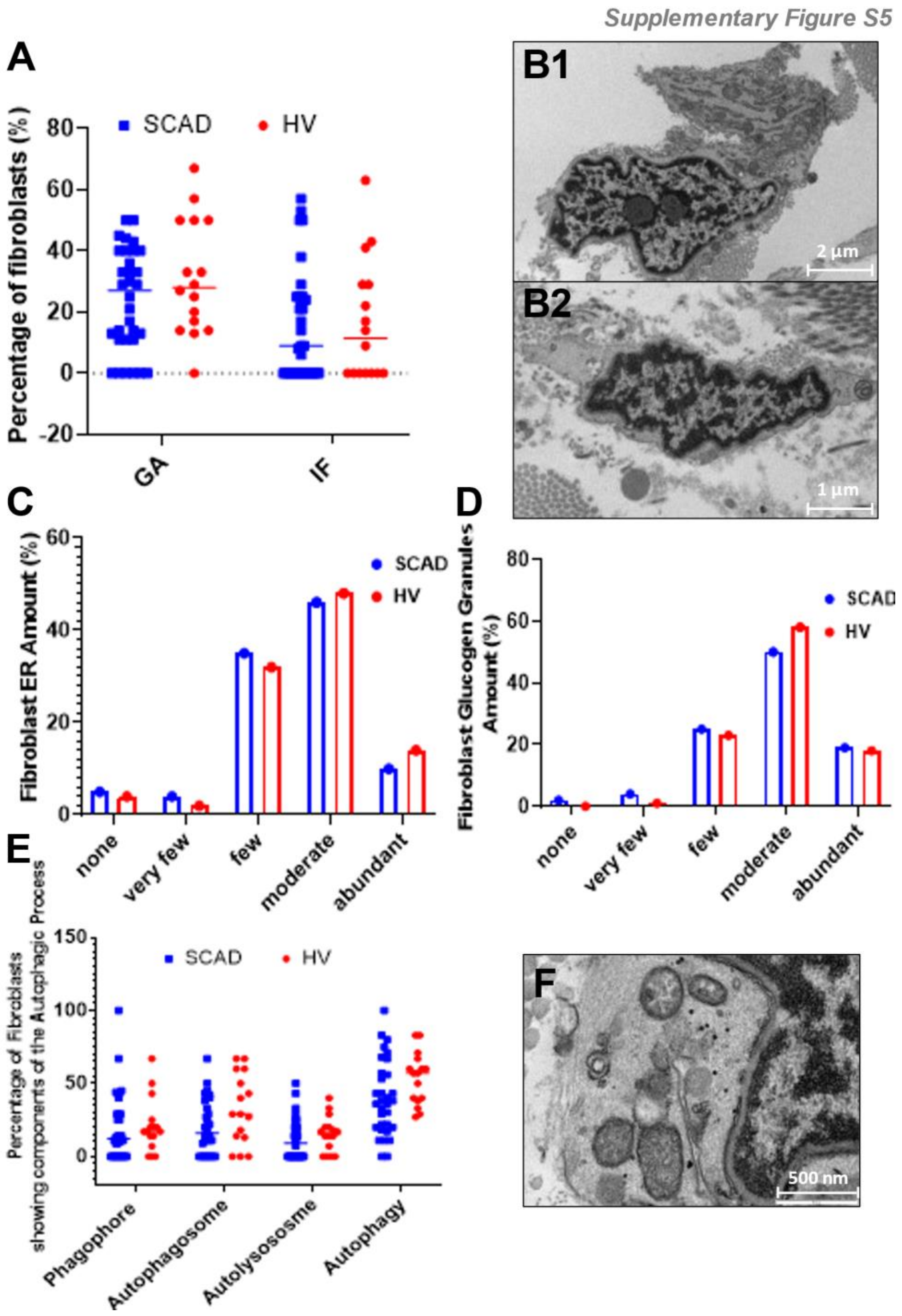

Supplementary Figure S5. Fibroblast components and activity.

Panel A: In order to assess the balance between quiescence and cell activity in skin fibroblasts isolated from Healthy Volunteers (HV, n=16) versus SCAD survivors(n=31), the presence of Golgi Apparatus (GA) and Intermediate Filaments (IF) was estimated using electron microscopy and presented as percentage of fibroblasts. Panel B1 and B2: examples of collagen granules and endoplasmic reticulum in fibroblast cells observed at the TEM. Mann-Whitney U-test was performed to compare SCAD and HV groups for these cell components. No significant differences were observed. Panels C and D show the percentage of fibroblasts with different amounts of endoplasmic reticulum and glycogen granules. Chi-square test was performed between the groups shown. No significant differences were observed. Panel E: Comparison of percentage of fibroblasts that showed presence of the main components of the Autophagic process (Phagophore, Autophagophore, Autolysosome, Autophagy) between SCAD and HVs. Panel F: fibroblast cytoplasm with glycogen granules and autophagophore. Two-Way ANOVA did not show significant differences between the two populations.

Supplementary Figure S6

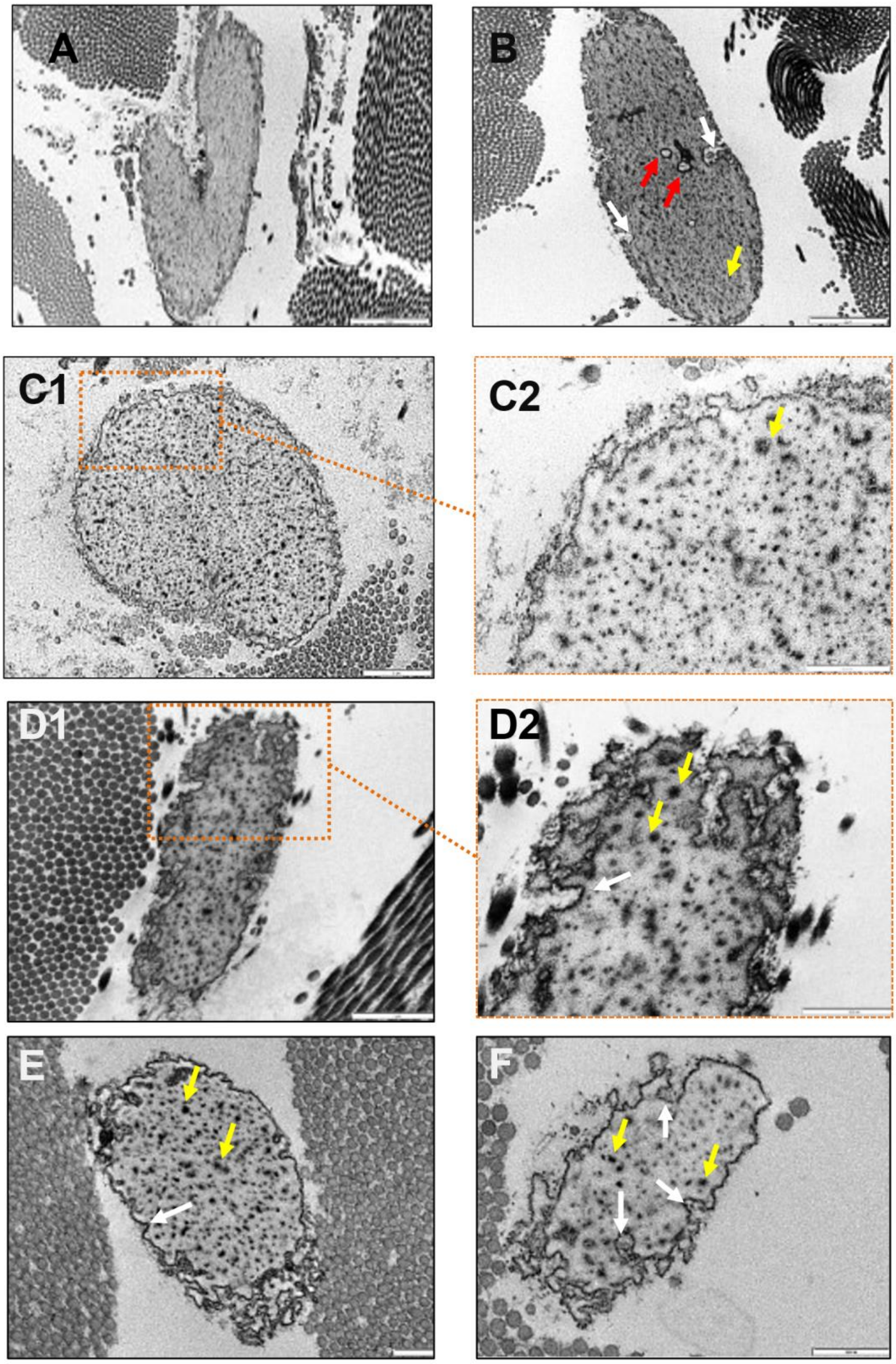

Supplementary Figure S6: Elastin feature analyses.
Panel A: Example of normal elastin with normal edges and no dense spots or indentations. Panels B, C1 & C2: Examples of elastin with moderately frayed edges, dense internal spots (yellow arrows), indentations (white arrow) and calcified microcavities (red arrows). Panel C2 is a detail of Panel C1. Panels D, E & F: Examples of elastin with moth eaten edges and thick surface coat either in SCAD patients (Panels D1&D2) or healthy volunteers (Panel E&F). Both show indentations (white arrows) and dense internal spots (yellow arrows). Panel D2 is a detail of D1.

Supplementary Figure 7. STROBE Statement—checklist of items that should be included in reports of observational studies

	Item No	Recommendation	Page No
**Title and abstract**	1	(*a*) Indicate the study's design with a commonly used term in the title or the abstract	N/A
		(*b*) Provide in the abstract an informative and balanced summary of what was done and what was found	2
**Introduction**			
Background/rationale	2	Explain the scientific background and rationale for the investigation being reported	4
Objectives	3	State specific objectives, including any prespecified hypotheses	4-5
**Methods**			
Study design	4	Present key elements of study design early in the paper	5
Setting	5	Describe the setting, locations, and relevant dates, including periods of recruitment, exposure, follow-up, and data collection	5
Participants	6	(*a*) *Cohort study*—Give the eligibility criteria, and the sources and methods of selection of participants. Describe methods of follow-up<br>*Case-control study*—Give the eligibility criteria, and the sources and methods of case ascertainment and control selection. Give the rationale for the choice of cases and controls<br>*Cross-sectional study*—Give the eligibility criteria, and the sources and methods of selection of participants	5
		(*b*) *Cohort study*—For matched studies, give matching criteria and number of exposed and unexposed<br>*Case-control study*—For matched studies, give matching criteria and the number of controls per case	5
Variables	7	Clearly define all outcomes, exposures, predictors, potential confounders, and effect modifiers. Give diagnostic criteria, if applicable	5-8
Data sources/ measurement	8*	For each variable of interest, give sources of data and details of methods of assessment (measurement). Describe comparability of assessment methods if there is more than one group	6-8
Bias	9	Describe any efforts to address potential sources of bias	N/A
Study size	10	Explain how the study size was arrived at	5-6
Quantitative variables	11	Explain how quantitative variables were handled in the analyses. If applicable, describe which groupings were chosen and why	8-9
Statistical methods	12	(*a*) Describe all statistical methods, including those used to control for confounding	8-9
		(*b*) Describe any methods used to examine subgroups and interactions	N/A
		(*c*) Explain how missing data were addressed	N/A
		(*d*) *Cohort study*—If applicable, explain how loss to follow-up was addressed<br>*Case-control study*—If applicable, explain how matching of cases and controls was addressed<br>*Cross-sectional study*—If applicable, describe analytical methods taking account of sampling strategy	5
		(*e*) Describe any sensitivity analyses	N/A

Continued on next page

**Results**			
Participants	13*	(a) Report numbers of individuals at each stage of study—eg numbers potentially eligible, examined for eligibility, confirmed eligible, included in the study, completing follow-up, and analysed	9-10
		(b) Give reasons for non-participation at each stage	N/A
		(c) Consider use of a flow diagram	Figure S1
Descriptive data	14*	(a) Give characteristics of study participants (eg demographic, clinical, social) and information on exposures and potential confounders	Tables 1-3
		(b) Indicate number of participants with missing data for each variable of interest	N/A
		(c) *Cohort study*—Summarise follow-up time (eg, average and total amount)	N/A
Outcome data	15*	*Cohort study*—Report numbers of outcome events or summary measures over time	N/A
		Case-control study—Report numbers in each exposure category, or summary measures of exposure	Figures S5/S6
		Cross-sectional study—Report numbers of outcome events or summary measures	N/A
Main results	16	(*a*) Give unadjusted estimates and, if applicable, confounder-adjusted estimates and their precision (eg, 95% confidence interval). Make clear which confounders were adjusted for and why they were included	9-15
		(*b*) Report category boundaries when continuous variables were categorized	Tables S1&S2
		(*c*) If relevant, consider translating estimates of relative risk into absolute risk for a meaningful time period	N/A
Other analyses	17	Report other analyses done—eg analyses of subgroups and interactions, and sensitivity analyses	Tables S1&S2
**Discussion**			
Key results	18	Summarise key results with reference to study objectives	15-16
Limitations	19	Discuss limitations of the study, taking into account sources of potential bias or imprecision. Discuss both direction and magnitude of any potential bias	19
Interpretation	20	Give a cautious overall interpretation of results considering objectives, limitations, multiplicity of analyses, results from similar studies, and other relevant evidence	19
Generalisability	21	Discuss the generalisability (external validity) of the study results	19
**Other information**			
Funding	22	Give the source of funding and the role of the funders for the present study and, if applicable, for the original study on which the present article is based	20

*Give information separately for cases and controls in case-control studies and, if applicable, for exposed and unexposed groups in cohort and cross-sectional studies.
